# Effects of Peptide on NK Cell-Mediated MHC I Recognition

**DOI:** 10.3389/fimmu.2014.00133

**Published:** 2014-03-31

**Authors:** Sorcha A. Cassidy, Kuldeep S. Cheent, Salim I. Khakoo

**Affiliations:** ^1^Division of Medicine, Imperial College London, London, UK; ^2^Clinical and Experimental Sciences, Faculty of Medicine, Southampton General Hospital, University of Southampton, Southampton, UK

**Keywords:** KIR, NKG2A, CD94, peptides, MHC class I, natural killer cells, antagonism, synergy

## Abstract

The inhibitory receptors for MHC class I have a central role in controlling natural killer (NK) cell activity. Soon after their discovery, it was found that these receptors have a degree of peptide selectivity. Such peptide selectivity has been demonstrated for all inhibitory killer cell immunoglobulin-like receptor (KIR) tested to date, certain activating KIR, and also members of the C-type lectin-like family of receptors. This selectivity is much broader than the peptide specificity of T cell receptors, with NK cell receptors recognizing peptide motifs, rather than individual peptides. Inhibitory receptors on NK cells can survey the peptide:MHC complexes expressed on the surface of target cells, therefore subsequent transduction of an inhibitory signal depends on the overall peptide content of these MHC class I complexes. Functionally, KIR-expressing NK cells have been shown to be unexpectedly sensitive to changes in the peptide content of MHC class I, as peptide:MHC class I complexes that weakly engage KIR can antagonize the inhibitory signals generated by engagement of stronger KIR-binding peptide:MHC class I complexes. This property provides KIR-expressing NK cells with the potential to recognize changes in the peptide:MHC class I repertoire, which may occur during viral infections and tumorigenesis. By contrast, in the presence of HLA class I leader peptides, virus-derived peptides can induce a synergistic inhibition of CD94:NKG2A-expressing NK cells through recruitment of CD94 in the absence of NKG2A. On the other hand, CD94:NKG2A-positive NK cells can be exquisitely sensitive to changes in the levels of MHC class I. Peptide antagonism and sensitivity to changes in MHC class I levels are properties that distinguish KIR and CD94:NKG2A. The subtle difference in the properties of NK cells expressing these receptors provides a rationale for having complementary inhibitory receptor systems for MHC class I.

## Introduction

Our knowledge of the functional role of natural killer (NK) cells has greatly increased in recent years. Originally thought to mainly recognize infected or neoplastic cells, NK cells are now known to help shape the adaptive immune response through direct interactions with dendritic cells, macrophages, and T cells (1, 2). NK cells integrate the signals derived from cellular contacts to determine whether or not effector functions are initiated. Due to a dominance of inhibitory over activating signals, healthy or quiescent cells do not activate NK cells. The ability to recognize changes in target cell state has been related to up-regulation of ligands for activating receptors (“induced self-recognition”), and down-regulation of ligands for inhibitory receptors (“missing self-recognition”) (3, 4). In humans, the most important inhibitory receptors for MHC class I comprise molecules from the killer cell immunoglobulin-like receptor (KIR) or C-type lectin-like receptor (CD94:NKG2A) families.

The inhibitory KIR recognize specific HLA-A, -B, and -C alleles. In particular, KIR3DL2 binds HLA-A*03 and HLA-A*11; KIR3DL1 binds HLA-B alleles with the Bw4 serological motif; KIR2DL1 binds HLA-C alleles with lysine at position 80 (group 2 HLA-C); and both KIR2DL2 and KIR2DL3 bind HLA-C alleles with asparagine at position 80 (group 1 HLA-C alleles) (5). Thus, it was originally considered that simple structural motifs determined the engagement of KIR with MHC. However, detailed analysis of KIR binding has shown that KIR2DL2 can bind the recombinant HLA-B*4601 and B*7301 alleles, which have HLA-C-type motifs at residues 77–83 (6). Furthermore, KIR2DL2 can interact with a number of group 2 HLA-C alleles, as can KIR2DL3, albeit to a lesser extent, as the affinity of KIR2DL3 for MHC is lower than that of KIR2DL2 (7–9). Therefore, although motifs at residues 77–83 appear to dominate the specificity of the interaction between KIR and MHC, it is clear that these effects can be modified by additional contacts between KIR and the MHC class I heterotrimer.

## Peptide Selectivity of Inhibitory Receptors

Key experiments performed in the mid 1990s demonstrated that the KIR are sensitive to the peptide bound by MHC class I. This was originally shown for KIR3DL1 and HLA-B*2705, and then for KIR2DL1 (10–12). Subsequent work extended these findings to KIR2DL2, KIR2DL3, and KIR3DL2 (13–18). These functional experiments are supported by co-crystal structures of KIR and MHC class I. The co-crystal of KIR2DL2 and HLA-Cw*03 with the GAVDPLLAL peptide demonstrated that specific residues of KIR directly contact P7 and P8 residues of the bound peptide (13). Similarly, P8 of the LSSPVTKSF peptide in HLA-B*5701 contacts residue 166 of KIR3DL1 (19). In the crystal structure of KIR2DL1 with HLA-Cw4, direct contacts between KIR and MHC class I peptide are not observed (20). Nevertheless, P8 is solvent accessible and changes in this residue do lead to alterations in NK cell function, implying secondary effects of MHC class I peptide on KIR2DL1:HLA-C binding.

The C-type lectin-like receptor NKG2A forms a heterodimer with a related family member CD94 to recognize the non-classical MHC class I molecule HLA-E (21–25). In general, this molecule binds leader peptides derived from HLA-A, -B, and -C molecules (26). Inhibitory signaling by CD94:NKG2A is also critically dependent on the peptide presented by HLA-E, and a hierarchy of HLA-E-binding peptides with different inhibitory properties for NKG2A-positive NK cells has been established (27–29). The peptide dependence of CD94:NKG2A was confirmed in its co-crystal structure with HLA-E and the HLA-G leader peptide VMAPRTLFL (30, 31). These studies showed that binding of CD94:NKG2A is dominated by the non-signaling CD94 moiety, and crucially P5, P6, and P8 have contacts with CD94 and P8 contacts NKG2A. The importance of these specific residues has been confirmed in surface plasmon resonance studies (32). Therefore, both inhibitory KIR and NKG2A are peptide selective. Furthermore, despite the rapid evolution of the KIR alongside that of classical MHC class I, this peptide dependence is a feature that has been retained across divergent KIR lineages (33).

## Peptide Antagonism of KIR-Positive NK Cells

Using a model system, we have investigated the functional consequences of KIR peptide selectivity. The T2 and 721.174 cell lines both synthesize HLA-Cw*0102 but have lost the ability to load peptide onto MHC due to a deficiency in transporter associated with antigen processing (TAP) (34). In these cell lines, MHC class I contains low affinity hydrophobic peptides derived from signal sequences, and reaches the cell surface but dissociates rapidly (35, 36). These TAP-deficient cell lines can be readily loaded with exogenous peptide as was originally demonstrated in CTL cell assays. HLA-Cw*0102 is of the HLA-C1 group specificity and hence engages with KIR2DL2 and KIR2DL3. These cell lines have allowed detailed examination of how KIR-positive NK cells respond to changes in the peptide content of MHC class I.

**Figure 1:**
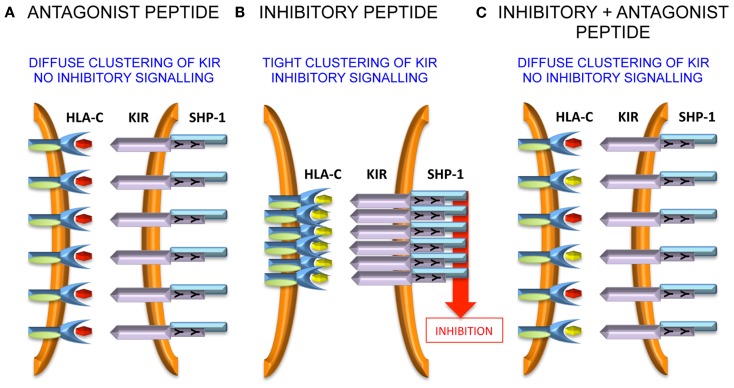
**Peptide antagonism is associated with loss of tight clustering of KIR**. **(A)** The antagonist peptide induces clustering of KIR and SHP-1 recruitment to the immune synapse. However, the clustering is diffuse and there is no inhibitory signal generated. **(B)** An inhibitory peptide induces tight clustering of KIR, SHP-1 recruitment, and inhibitory signaling. **(C)** The antagonist peptide abrogates tight clustering and thence inhibitory signaling.

VAPWNSLSL is a peptide derived from TIMP1 that was eluted from an HLA-Cw*0102 transfectant of the MHC class I deficient 721.221 cell line (37). This peptide was used as a backbone to screen derivatives that differ only at the KIR-binding residues of P7 and P8 (38). Overall, although all peptides stabilized HLA-Cw*0102, in assays using KIR-fusion constructs only approximately a third of the peptides induced significant binding to KIR2DL2 and KIR2DL3. This allowed the definition of strong, weak, and null KIR binders, and this binding reactivity correlated well with their inhibitory potential in assays of NK cell function. We used the strongest inhibitory peptide VAPWNS*F*AL (VAP-FA), and the non-inhibitory peptide VAPWNS*D*AL (VAP-DA) to study how NK cells may respond to changes in peptide repertoire (38). Although it would be predicted that VAP-DA would have a null effect on the inhibition due to VAP-FA, it was found to modulate the inhibition of KIR2DL2/3-positive NK cells. We termed this phenomenon “peptide antagonism” to indicate that peptides that act alone have no effect on NK cell function, can modulate the inhibition due to inhibitory peptides. The mechanism for peptide antagonism may be related to a low affinity interaction between KIR and peptide:MHC. This phenomenon was confirmed using a tyrosine P8 substitution in VAP-DA, as tyrosine P8 substantially reduces binding of KIR2DL2 to HLA-Cw*03 in surface plasmon resonance studies (13). Further studies have shown that although VAP-DA does not bind KIR in the fusion construct assay, it can induce diffuse clustering of KIR2LD3 at the interface between NK cells and 721.174 cells (39). Additionally, it induces recruitment of SHP-1 to KIR2DL3, but it abrogates the formation of KIR2DL3 microclusters by VAP-FA, and thus it prevents downstream inhibitory signaling (Figure 1).

The precise molecular mechanism of antagonism is not clear at present (40). It appears to be dependent on the presence of immunoreceptor tyrosine-based inhibitory motifs (ITIMs), as in their absence there is no effect of the VAP-DA peptide on the microcluster formation by VAP-FA, although notably much lower levels of microcluster formation are observed in these experiments (39). Therefore, intracellular signaling events are likely to be important. It has been shown that rearrangement of the actin cytoskeleton is important for KIR accumulation at the NK cell synapse and it may be that this rearrangement is impaired by antagonist peptides (41). SHP-1 requires tyrosine phosphorylation for full activation (42). Therefore, although SHP-1 may be recruited by VAP-DA, it may not be phosphorylated and so not fully activated so that downstream inhibitory signaling events remain uninitiated. Alternatively, the low affinity KIR:HLA-C:VAP-DA complexes could rapidly dissociate before productive inhibitory clustering has taken place. By recruiting SHP-1 to these transient complexes, it could sequester SHP-1 away from the more stable KIR:HLA-C:VAP-FA complexes and thus prevent an inhibitory signal being generated. These events require additional investigation to determine the precise mechanisms governing the phenomenon of “peptide antagonism.”

## Peptide Synergy of NKG2A-Positive NK Cells

In contrast to the KIR, the C-type lectin-like receptors are relatively conserved in terms of evolution. The human receptor binds the non-classical HLA-E molecule which in healthy cells presents peptides derived from the leader sequences of other MHC class I molecules, including HLA-A, -B, -C, and -G (26). A homologous receptor:ligand partnership is present in the mouse.

Murine CD94:NKG2A interacts with the non-classical Q-a1 molecule, which also binds a leader sequence, Qdm, derived from MHC class I (43–45). In the absence of TAP, HLA-E has been shown to bind a wider variety of peptides (46), and can also bind viral peptides derived from CMV (VMAPRTLIL), EBV (SQAPLPCVL), HIV (AISPRTLNA), and HCV (YLLPRRGPRL) (47–50). In terms of peptide sequence, the CMV peptide is derived from the signal sequence of UL-40 and the common variant is identical to the HLA-Cw*03 leader sequence (51). The peptides from EBV, HIV, and HCV have less sequence homology to MHC class I leader peptides and were identified by functional approaches. Detailed investigation of the HCV core_35–44_ peptide YLLPRRGPRL demonstrated that although it stabilized HLA-E on the surface of the TAP-deficient 721.174 cell line, these peptide-loaded targets did not inhibit NKG2A-positive NK cells (52). However, it was noted that relatively small amounts of leader sequences derived from HLA-A, -B, and -G could inhibit a fraction of NKG2A-positive NK cells and that addition of HCV core_35–44_ increased that inhibition. This was also true for the HIV and EBV-derived peptides as well as the Hsp60 leader peptide, which had previously been shown to bind to HLA-E and engage the activating receptor NKG2C. In experiments studying the clustering of CD94:NKG2A at the interface between NK cells and peptide-loaded 721.174 cells, it could be demonstrated that HCV core_35–44_ induced clustering of CD94, but not of NKG2A. Furthermore, this clustering could be abrogated by mutating P5 of the peptide from arginine to lysine, a substitution that would be predicted to prevent binding to CD94. As CD94 can exist on the cell surface as a homodimer, we proposed that the HLA-E:YLLPRRGPRL complex engages the CD94 homodimer, but not the CD94:NKG2A heterodimer (53, 54). Although CD94 does not have a signaling motif in its cytoplasmic tail and is not thought to mediate signaling on its own, stabilization of CD94 homodimers could lead to higher order receptor clustering and augment inhibitory signaling in NKG2A-positive NK cells. Additionally, an HLA-E tetramer loaded with the HLA-Cw*03 peptide VMAPRTLIL binds well to NKL which express CD94:NKG2A heterodimers, but not to Jurkat cells expressing only CD94. This, combined with the functional data, indicates that CD94:NKG2A and CD94 homodimers have different peptide specificities.

**Figure 2:**
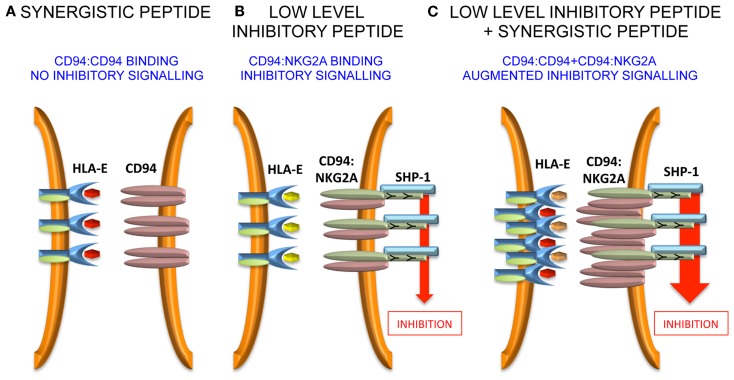
**Model for peptide synergy**. **(A)** A synergistic peptide induces recruitment of CD94 homodimers, but not CD94:NKG2A to the immune synapse. Therefore, there is no inhibitory signal generated. **(B)** At low levels of inhibitory peptide CD94:NKG2A is recruited to the immune synapse and there is inhibitory signaling. **(C)** The synergistic peptide augments the inhibitory signaling due to low levels of inhibitory peptide by stabilizing the immune synapse.

Further work needs to be performed to define precisely how the “non-signaling” CD94 molecule influences inhibitory signaling. Unlike antagonism for KIR, it is less likely that intracellular effects are important because CD94 possesses only a short intra-cytoplasmic tail, and is not thought to have a signaling function in isolation or in combination with a signaling adaptor protein. Therefore, extracellular effects may be relevant and one hypothesis could be that CD94 homodimers assist in the formation of macromolecular aggregates of the CD94:NKG2A heterodimer. Such aggregates may stabilize receptor:ligand contacts at the immune synapse and augment inhibitory signaling (Figure 2).

**Figure 3:**
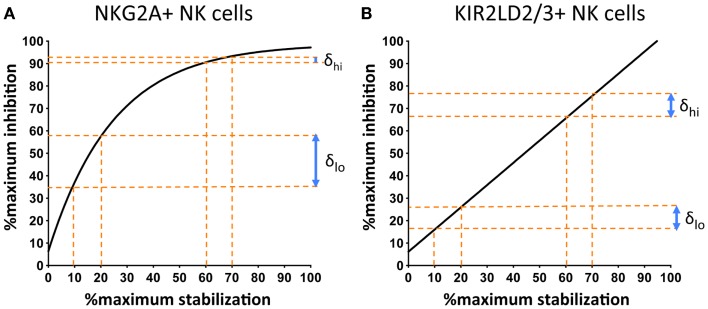
**NKG2A+ NK cells are more sensitive to changes in the cell-surface levels of MHC class I at low, as compared to high, surface levels of MHC class I**. 721.174 cells were loaded with increasing concentrations of peptide and used as targets in CD107a assays. The levels of degranulation of NKG2A+ **(A)** or KIR2D:2/3+ **(B)** NK cells were plotted against levels of cognate MHC class I:peptide. In **(A)** HLA-E was loaded with increasing concentrations of the HLA-G leader peptide and in **(B)** HLA-Cw*0102 was loaded with increasing concentrations of the VAP-FA peptide. At between 10 and 20% of maximal HLA-E levels, there is an increase of ~20% in the fraction of degranulating NKG2A+ NK cells (δ_lo_) but between 60 and 70% of maximal HLA-E levels, this change is less than 5% (δ_hi_). For KIR+ NK cells, δ_lo_ and δ_hi_ are similar at ~10%. Data were derived from Cheent et al. (47).

Comparison of the response of NKG2A+ and KIR+ NK cells to changes in cell-surface MHC class I demonstrate an additional important difference. The stoichiometry of KIR-mediated inhibition and MHC class I cell-surface expression is linear, whereas that of MHC class I with NKG2A exhibits saturation kinetics (Figure 3). This can be expressed as follows:

$${\text{Degranulation}}_{\left(\text{KIR}+\text{NK Cells}\right)}=k_{1}\left(\text{MHC I}\right)$$

but

$${\text{Degranulation}}_{\left(\text{NKG2A}+\text{NK Cells}\right)}=k_{2}\left(\text{MHC I}\right)∕\left[\text{x}+\left(\text{MHC I}\right)\right]$$

Thus, an additional factor “x” is required to explain the relationship of NKG2A-mediated degranulation to MHC class I cell-surface levels. This additional factor may be a constant or a variable. However, the key feature is that it is more dominant at low rather than high cell-surface MHC class I levels, thus reflecting the greater sensitivity of NKG2A-positive NK cells to changes in MHC class I cell-surface levels as compared to KIR-positive NK cells (Figure 3). One potential factor could be that CD94:NKG2A forms macromolecular aggregates, which are facilitated by CD94 homodimers. Conversely, the 2Ig domain KIR may not form aggregates spontaneously. Aggregation of KIR is a contentious issue. KIR binding to MHC is dependent on the presence of zinc ions, with KIR having a zinc-binding motif (55, 56). Furthermore, both zinc and cobalt can mediate aggregate formation *in vitro* (57, 58). In crystallographic studies of KIR2DL2 with HLA-Cw*03, KIR2DL2 have also demonstrated the formation of multimers and, based on this feature, a model for KIR aggregation was proposed (59, 60). Subsequently little additional data has been generated on this model and its *in vivo* significance is less clear. Conversely for the 3Ig domain KIR, the D0 domain appears to assist in signaling, even though it does not contact its MHC class I ligand (19, 61). Therefore, it has been proposed that the D0 domain assists in aggregation of KIR. The ability to form, or not to form, multi-molecular aggregates may be relevant to the differences we have observed in how KIR- and NKG2A-positive NK cells respond to changes in peptide. However, clearly these mechanisms require additional structural and functional investigation.

## Considerations for Viral Infections

As both KIR and CD94:NKG2A are peptide selective receptors, this implies that NK cells may be sensitive to changes in the peptide repertoire presented by MHC class I. Therefore, the content and economics of peptide presentation is a key consideration in determining if this could be a feasible mechanism for changing NK cell reactivity.

The MHC class I peptidome is a complex mixture of host peptides. The MHC class I repertoire on the cell surface is the result of several processes: cellular protein degradation; access of peptides to nascent MHC class I molecules; and the multi-step process of peptide loading. Viral infection can alter this at many levels, including switching off host protein synthesis, turning on viral peptide synthesis, interfering with MHC class I peptide loading, and changing the recycling of MHC class I, leading to cell-surface down-regulation (62).

Until recently, it was thought that peptides presented by MHC class I were derived from the degradation of mature proteins or “retirees.” However, there can be marked changes in the efficiency by which specific peptide epitopes are generated (63–65) and recent data suggest that a substantial fraction of MHC class I peptide derives from defective protein synthesis or “DRiPs” (defective ribosomal products) (66, 67). Up to 70% of proteins synthesized may be degraded before forming functional proteins as the result of defective transcription, failed assembly, mistakes by amino-acyl t-RNA synthetases, or altered ubiquitin modifications (68).

DRiPs may additionally be derived from alternative open-reading frames, and the presentation of these “cryptic” epitopes may make understanding the peptide repertoire more difficult (69). In a system in which the MHC class I peptidome is derived from mature proteins, the turnover and abundance of cellular proteins will determine the nature of peptides presented. However, in the case of DRiPs, this becomes less predictable and the peptide repertoire becomes determined by both mRNA abundance and also error rates in protein synthesis. Errors in protein transcription that ultimately lead to proteins with aberrant sequences are more likely to be more common for viral, as opposed to host proteins, as viral RNA polymerases may lack proof reading capacity. Thus, for the HCV RNA dependent-RNA polymerase, estimated error rates may be as high as one per 1000 per nucleotide site (70). As the HCV genome is only 9.3 kb long, there is a substantial probability of mutation, which on the one hand favors viral escape mutation, but may also lead to the synthesis of DRiPs. Favoring the DRiPs model, viral epitopes for CTL have been shown to be generated from recently synthesized peptides, rather than from mature proteins, confirming the potential of this mechanism for altering the host peptide repertoire (71). Additionally, the efficiency of presentation of an epitope may depend on the source, viral or cellular, of the mRNA and there may also be compartmentalization in the subcellular localization of peptides for class I presentation (65, 72).

Thus, generating a peptide repertoire in the context of a viral infection is a complex procedure that is not readily predictable. Analysis of the MHC class I peptidome reveals that after HIV infection the majority of peptides are self-peptides (73). Conversely, in some infections, there can be substantial numbers of viral peptides presented by MHC class I. For instance, in measles virus infection, the HLA-A*0201 epitope KLWESPQEI epitope has been suggested to be as abundant as 5 × 10^4^ copies per cell (74). Quantitation of viral epitopes is therefore a key factor, as although both KIR-positive and CD94:NKG2A-positive NK cells are sensitive to changes in peptide repertoire, the relative magnitude of these changes will likely be important. Additionally, it has been shown that KIR2DL2 can be a driving force on HIV sequence (75) and the selection of a strong inhibitory peptide may “tip the balance” in terms of evasion of the immune response by the virus. However, accurate quantitation is required to determine whether this is due solely to viral peptides or a combination of host and viral peptides. Indeed, the broad peptide specificity of KIR implies that host peptides would be as effective as viral peptides in altering NK cell reactivity. Interference with host protein synthesis by virus infection may enhance the formation of DRiPs that could then lead on to large changes in peptide repertoire (76, 77). Furthermore, a hold-up in protein degradation can feedback negatively on protein synthesis and translation, additionally modifying the peptides available for presentation by MHC class I (78). Thus, formation of a peptide repertoire, and how a virus interferes with it, is a complex procedure, which at present requires much more detailed understanding before we can learn how this can impact inhibitory receptor signaling by NK cells.

A number of key questions remain to be answered with respect to peptide antagonism. At present, this phenomenon has only been demonstrated for one receptor:ligand system, and whether this extends to other KIR, or even KIR2DL2/3 with other group 1 HLA-C ligands needs to be examined. When the breadth of peptide antagonism is understood, then it will be possible to determine the physiological relevance of it for viral infections, and in particular how commonly antagonism affects the balance between inhibition and activation of an NK cell in physiological and pathological situations. Furthermore, individual peptides will need to be examined in greater detail to understand precisely which peptides are antagonistic and how this correlates with binding. Comparing peptides eluted from group 1 HLA-C molecules as described in the SYFPEITHI database (79), with binding studies using KIR2DL2 fusion constructs (38) suggests that about half of these peptide are unlikely to bind KIR, which speculatively would correlate with the number considered to be antagonist. This estimate has the condition that at present we do not know the limits of binding-affinity to KIR for antagonist peptides; that is at which point a peptide has a high enough affinity to act as a weak binder or conversely an affinity so low that it may be null. This may be determined by the overall binding of the peptide:MHC complex for KIR, rather than just the peptide, so different HLA-C alleles may have different frequencies of peptides that fall into the inhibitory, antagonistic, and null categories. One study in HIV has shown that the majority of peptides is non-KIR binders and hence could fall into the antagonistic or null categories (80). In this work of 217 HIV-derived peptides tested, 11 were identified that bound HLA-Cw*0102, and only one of these bound KIR2DL2.

## Subtly Different Functions of KIR and NKG2A

For CD94:NKG2A, the broadening in peptide specificity afforded by engagement of CD94 homodimers could be exploited by viruses, to augment inhibition of NKG2A-positive NK cells. This contrasts with the observations for KIR, as to date we have found that peptides engaging KIR2DL2/3 that do not inhibit directly, can perturb inhibitory the signaling generated by high affinity KIR:MHC:peptide complexes. As the majority of peptide variants that we tested are non-KIR binders, this suggests that changes in peptide repertoire that affect KIR are more likely to result in loss of inhibition. This raises the possibility that KIR and CD94:NKG2A may have subtly different functions. As discussed above, NKG2A-positive and KIR2DL2/3-positive NK cells respond with different stoichiometries to changes in the levels of cell-surface MHC class I. These data imply that NKG2A-positive NK cells are exquisitely sensitive to changes in MHC class I cell-surface levels at low levels of MHC class I, in our peptide titration experiments at <1% of maximal cell-surface levels. We thus propose that NKG2A is a receptor well adapted to changes in the cell-surface quantity of MHC class I. Conversely, KIR are not specialized for this function but may be more sensitive to changes in peptide repertoire. It has been proposed that KIR have a specialization to recognize cells that have down-regulated specific HLA-A, -B, -C molecules. However, as most HLA molecules have leader peptides cognate for HLA-E and CD94:NKG2A, then NKG2A-positive NK cells would serve this function adequately and, at low levels of MHC class I, most likely better than KIR. We propose that the HLA-C specific KIR are specialized to detect changes in peptide repertoire and that this function complements the role of NKG2A in detecting MHC class I down-regulation. If KIR-positive and NKG2A-positive NK cells have these subtly different functions *in vivo* then this would provide a rationale for having two distinct inhibitory receptor systems for MHC class I.

## Conclusion

In depth study of the peptide selectivity of KIR2DL2/3 and CD94:NKG2A have given novel insights into the functions of these receptors. In addition to induced self- and missing self-recognition, it may be that an “altered self-recognition” is also important for NK cells expressing these receptors. Testing of these models *in vivo* is now required to establish the significance of these observations for disease.

## Conflict of Interest Statement

The authors declare that the research was conducted in the absence of any commercial or financial relationships that could be construed as a potential conflict of interest.
